# Magnetisation Processes in Geometrically Frustrated Spin Networks with Self-Assembled Cliques

**DOI:** 10.3390/e22030336

**Published:** 2020-03-14

**Authors:** Bosiljka Tadić, Miroslav Andjelković, Milovan Šuvakov, Geoff J. Rodgers

**Affiliations:** 1 Department of Theoretical Physics, Jožef Stefan Institute, SI 1000 Ljubljana, Slovenia; 2 Complexity Science Hub Vienna, Josephstädter Strasse 39, A 1080 Vienna, Austria; 3 Institute for Nuclear Sciences Vinča, University of Belgrade, 11000 Belgrade, Serbia; mandjelkovic@vin.bg.ac.rs; 4 Institute of Physics Belgrade, University of Belgrade, Pregrevica 118, 11080 Belgrade, Serbia; suvakov@gmail.com; 5 Department of Health Sciences Research, Center for Individualized Medicine, Mayo Clinic, Rochester, MN 55905, USA; 6 Department of Mathematics, Brunel University London, Uxbridge Middlesex UB8 3PH, UK; G.J.Rodgers@brunel.ac.uk

**Keywords:** spin dynamics, nanonetworks, simplex aggregation, hysteresis, antiferromagnetic defects

## Abstract

Functional designs of nanostructured materials seek to exploit the potential of complex morphologies and disorder. In this context, the spin dynamics in disordered antiferromagnetic materials present a significant challenge due to induced geometric frustration. Here we analyse the processes of magnetisation reversal driven by an external field in generalised spin networks with higher-order connectivity and antiferromagnetic defects. Using the model in (Tadić et al. Arxiv:1912.02433), we grow nanonetworks with geometrically constrained self-assemblies of simplexes (cliques) of a given size *n*, and with probability *p* each simplex possesses a defect edge affecting its binding, leading to a tree-like pattern of defects. The Ising spins are attached to vertices and have ferromagnetic interactions, while antiferromagnetic couplings apply between pairs of spins along each defect edge. Thus, a defect edge induces n−2 frustrated triangles per *n*-clique participating in a larger-scale complex. We determine several topological, entropic, and graph-theoretic measures to characterise the structures of these assemblies. Further, we show how the sizes of simplexes building the aggregates with a given pattern of defects affects the magnetisation curves, the length of the domain walls and the shape of the hysteresis loop. The hysteresis shows a sequence of plateaus of fractional magnetisation and multiscale fluctuations in the passage between them. For fully antiferromagnetic interactions, the loop splits into two parts only in mono-disperse assemblies of cliques consisting of an odd number of vertices *n*. At the same time, remnant magnetisation occurs when *n* is even, and in poly-disperse assemblies of cliques in the range n∈[2,10]. These results shed light on spin dynamics in complex nanomagnetic assemblies in which geometric frustration arises in the interplay of higher-order connectivity and antiferromagnetic interactions.

## 1. Introduction

Connectivity beyond standard pairwise interactions can be described by simplexes (cliques) of different orders; they constitute, for example, of groups of the system’s elements or vertices of the underlying network that join together to make organised complexes at a larger scale. These higher-order structures are found and are increasingly recognised as responsible for the performance of complex materials [1,2,3] and many complex systems from the brain [4,5,6] to large-scale social dynamics [7,8]. However, the mechanisms by which the architecture of simplexes that represent a particular physical system influences the dynamics of its units and shapes its global behaviour remain elusive. Recently, the focus of investigations of these systems has been diverted towards the features of their geometry that enables higher-order interactions among dynamical units [9,10,11,12,13,14,15].

In the framework of materials research, the cooperative self-assembly of nanoparticles, where preformatted groups of particles join a growing assembly, is a promising way to grow nanostructured materials of a complex morphology and improved functionality [16,17,18,19,20,21]. These magnetic materials, being assembled at the nanoscale, are quite different from conventional crystalline structures. Their distinctive features are, for example, the absence of apparent symmetry, and a number of closest neighbours that can vary in a wide range over the system. Mathematical modelling of the growth and architecture of these complex nanonetworks [22] reveals their higher organised structures [1,2,3,23,24]. These complex geometries can be described by simplicial complexes [3] within the algebraic topology of graphs [25]; they often have a negative curvature in the graph metric space [26,27]. Some examples that motivated our research include the soft magnetic materials and nanoassemblies that are relevant for a variety of applications [28,29]. Furthermore, such nanomagnetic materials enable the study of fundamental physics problems; in particular, the geometric frustration [30,31] and unusual magnetic order appearing in the presence of antiferromagnetic interactions [31,32,33,34,35,36,37]. The conditions where the geometric constraints combined with the sign of the interaction prevent simultaneous minimisation of the system’s global energy and each pair of spins lead to frustration [31]. A typical example is the case of Ising spins s=±1 on a triangular lattice when the combination of ferromagnetic/antiferromagnetic interactions $J_{ij}=\pm 1$ gives the product $J_{ij}J_{jk}J_{ki}<0$ along the triangle edges. In a more complex lattice geometry, different patterns of antiferromagnetic ordering take part, leading to the residual entropy [38] and a sequence of fractional magnetisation plateaus on the hysteresis loop in Ising-type [39,40,41] and XY-antiferromagnetic [42] systems.

In this work, we study the dynamics of spin systems on nanonetworks of the complex architecture of simplicial complexes, where the higher-order connectivity in conjunction with the antiferromagnetic defects give rise to geometric frustration. Based on the model of the self-assembly of simplexes with defect edges developed in [24], we grow different classes of nanonetworks, which enables us to explore the influences of their structures of simplicial complexes on the field-induced magnetisation processes they support. The Ising spins are associated with the network’s vertices; the spin kinetics are the subject of a pairwise ferromagnetic interaction along the edges of a simplex in the presence of an *antiferromagnetic spin–spin interaction along defect edges*. We apply the self-assembly rules in [24] by setting the chemical affinity parameter ν=0. Thus, the growth is governed by strictly geometrical compatibility of simplexes. As described below, a simplex of the size n=2,3,4,⋯ (i.e., a link, triangle, tetrahedron and so on) is formed by docking a growing nanonetwork, observing the geometric compatibility among the faces of glueing simplexes. Additionally, with probability *p*, each simplex can have a defect edge, which affects the rules, leading to a tree-like pattern of defects (see next section and [24]). Note that a simplex of the size *n* has (n3) triangles as its faces of the order q=2. Hence, each defect edge of a simplex of the size *n* induces n−2 frustrated triangles, which are part of a more massive structure. In the following, we show how the frustrated triangles influence the overall magnetisation process, depending on the size *n* of the elementary simplexes that make the assembly and the probability *p* that controls the formation of defect bonds. By varying the parameters *n* and *p*, we grow several classes of mono-disperse assemblies with n=3,4,⋯,7, and poly-disperse assemblies with n∈[2,10], and describe their structural features by graph-theoretic, algebraic topology and entropy measures. Further, we simulate the spin dynamics on them by slowly changing an external field along the hysteresis loop. We determine the magnetisation curves, the length of the domain walls and the appearance of the fractional-magnetisation plateaus, depending on the structural features of the assemblies and the present antiferromagnetic defects. For completeness, we also analyse the spin reversal dynamics in the fully antiferromagnetic and fully ferromagnetic limits.

## 2. Growth and Structures of Complex Assemblies

Following the original model of cooperative self-assembly developed in [3,24], here, we consider the geometrical assembly of simplexes that are full graphs (cliques) of *n* vertices in the presence of *defect edges*. The following rules govern the assembly process, starting with an initial clique. At each growth step, a new clique is added to the structure so that one of its geometrical faces is shared with an existing clique. The new clique has the specified size *n*, and with the probability *p*, one of the edges is a defect. The possible order of the geometrical faces is $q=0,1,2,3\cdots q_{max}-1$, where $q_{max}\equiv n-1$ is the order of the added clique. That is, the geometrically matching faces between the added and previously built-in cliques are found (see below), and the corresponding vertices that make that face are shared by docking the new clique along that face. Moreover, as explained in detail in the program flow Algorithm A1 in the Appendix A, the matching strictly observes the faces that contain a defect edge. Note that in the original model [3,24], the tuning towards large/small size of the shared face can be further affected by the chemical affinity ν of the assembly towards the number of added particles $n_{a}=q_{max}-q$. In the present work, we restrict the model to the geometrical compatibility rule only, corresponding to the case ν=0. Thus, the probability of the added clique of size *n* sharing a face of the order *q* along with the simplex σ of the order $q_{\sigma }=q$ reduces to [24],

(1) $$p_{\sigma }\left(q_{max},q;p,t\right)=\frac{c_{q}\left(p,t\right)}{\sum_{q=0}^{q_{max}-1}c_{q}\left(p,t\right)}\;.$$

**Figure 1:**
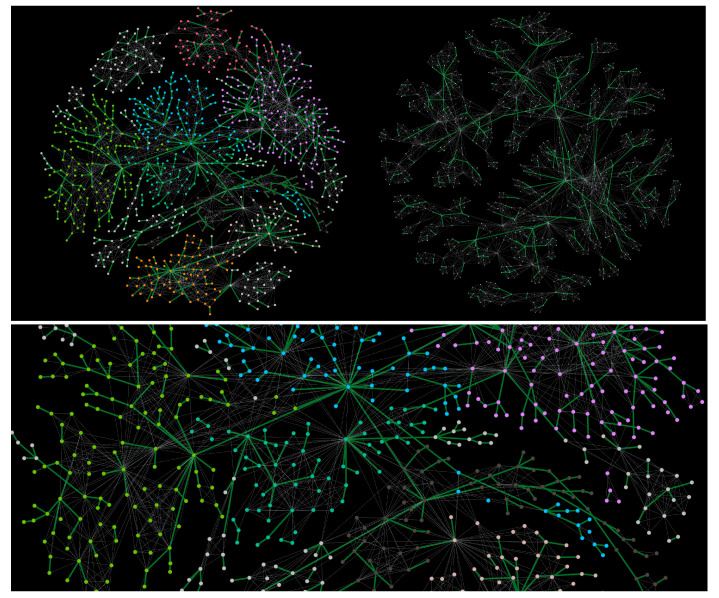
The assembly of the distributed simplex sizes n∈[2,10] according to $∼n^{-2}$ for strictly geometric aggregation (**top left**) and a close-up (**lower panel**); the assembly of mono-disperse simplexes of n=7 vertices (**top right**). One defect edge per simplex is present, shown as thick (green) lines; colours on nodes indicate mesoscopic communities.

Here $c_{q}\left(p,t\right)$ is the number of geometrically compatible locations on the growing structure at the time *t*. The parameter *p* in $c_{q}\left(p,t\right)$ indicates that the faces with a defect edge affect the matching possibilities for docking the corresponding face of the new clique. More specifically, it prevents faces with a defect edge from being shared with the faces with all straight edges of the new clique, while the defect edge of the new clique shares a defect vertex of the existing clique; see the program flow Algorithm A1. In analogy to the general model in [24], these assembly rules lead to a non-random pattern of defect edge. For this work, applying the probability in (1), we grow several mono-disperse assemblies of cliques of a given size n= 3, 4, 5, 6, and 7, and poly-disperse assemblies where n∈[2,10] is taken from the distribution $P_{n}∼n^{-2}$. Two representative examples in Figure 1 show the characteristic tree-like patterns of defect edges. The assemblies with mixed simplexes n∈[2,10] are investigated in detail by algebraic-topology techniques in [3,24] for pure and defect simplexes, respectively. The spectral analyses of the underlying topological graphs are given in [12]. The corresponding spin networks [43,44] are obtained by considering spins with two degrees of freedom attached to the vertices of these assemblies. Here we summarise some of their structural features that can be relevant to the geometric frustration in spin dynamics during the magnetisation reversal, studied in Section 3 and Section 4.

**Figure 2:**
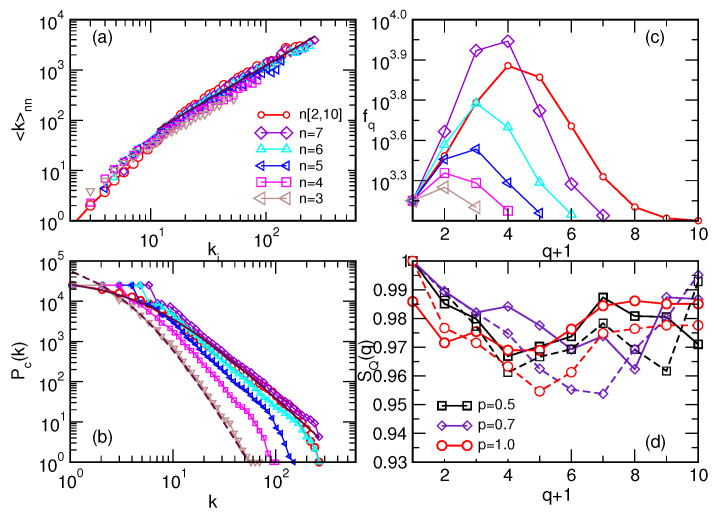
(**a**–**c**) Assortativity; cumulative distribution of degree (the number of nearest-neighbours); and geometric response $f_{q}$ for the poly-disperse assembly of simplexes of size n∈[2,10], and several mono-disperse assemblies of different simplex sizes *n* = 3, 4, 5, 6, and 7; the common legend is shown on panel (**a**). Panel (**d**) shows topological entropy $S_{Q}\left(q\right)$ against the topology level *q* for poly-disperse assembly n∈[2,10] for different probabilities of defect edges *p*, indicated in the legend of panel (**d**), and the corresponding assembly obtained by removing the defect edges.

The structural properties of all studied assemblies are summarised in Figure 2a–d. Specifically, these graphs exhibit a nontrivial correlation between the nodes expressed via assortative mixing. That is, the exponent μ>0 is found, connecting the degree of a node $k_{i}$ with the average degree of its nearest neighbours ${\langle k\rangle }_{nn}∼k_{i}^{\mu }$, for k≥10; cf. panel (a). The cumulative degree distribution in the panel (b) shows a broad range; depending on the size of the building simplexes, a region with a power-law decay occurs, as does a tendency towards “rich-club” grouping of the nodes of large degree, especially for larger *n*. The distribution of the assembly with mixed cliques interpolates between them; it fits with the Tsallis q−exponential distribution with the parameter $q_{Tsallis}=1.22$. The number of topology levels $q_{max}$ in these graphs is determined by the size of the largest clique built in the structure; i.e., $q_{max}+1=n$. For the composition of the assembly, it is relevant to determine how different vertices contribute to the structure seen at each topology level $q=0,1,2,\cdots q_{max}$. The number of simplexes and faces at each level *q*, denoted as $f_{q}$, is determined for each assembly and shown in Figure 2c. Assemblies with built-in larger cliques a have notably more abundant structure at intermediate *q* (levels) than the assemblies of mono-disperse cliques of that size. That is because higher-order cliques possess lower cliques of all orders, like their faces, which they can share by building the structure.

On the other hand, the presence of a large number of cliques of the same size, notably n=7, leads to a more productive structure regarding the connectivity (see panel (b)), which also has a more significant number of triangles and tetrahedra than the considered mixed assembly. These differences in the topology will have an impact on the spin dynamics, as we show in the following sections. Here, we also determine another topology measure of the mixed assemblies with defects; namely, the topological entropy measures $S_{Q}\left(q\right)$. Related to the probability $p_{i}\left(q\right)$ of the participation of a vertex *i* in the structures at the topology level *q*, the entropy is defined by the expression [45]

(2) $$S_{Q}\left(q\right)=-\frac{\sum_{i}p_{q}^{i}\log p_{q}^{i}}{\log M_{q}}\;.$$

Precisely, $p_{q}^{i}=\frac{Q_{q}^{i}}{\sum_{i}Q_{q}^{i}}$, where $Q_{q}$ indicates the *q*th component of the first structure vector; i.e., the number of q-connected classes. $M_{q}=\sum_{i}(1-\delta_{Q_{q}^{i},0})$ is the number of vertices with a non-zero entry at the level *q* in the entire graph; the sum is over all vertices. Figure 2d shows the topological entropy vs. *q* for the mixed assemblies grown at two different probabilities of defect edges p= 0.5 and 0.7. The dashed lines show how these entropy measures change when the structure of the assemblies simplifies by removing the defect bonds. Thus, in a particular way, these entropy measures quantify the importance of the defect edges in the actual structure. In the following sections, we will consider spin reversal dynamics on these assemblies assuming the antiferromagnetic defects along these defect edges.

## 3. Spin Dynamics and Hysteresis Loops in Different Assemblies

In the nanonetworks of self-assembled simplexes, the Ising spins $S_{i}=\pm 1$ are associated with the network vertices, and the field-driven dynamics of spins are governed by the following Hamiltonian.

(3) $$H=-\sum_{i,j}J_{ij}S_{i}S_{j}-h_{ext}\sum_{i}S_{i}\;.$$

**Figure 3:**
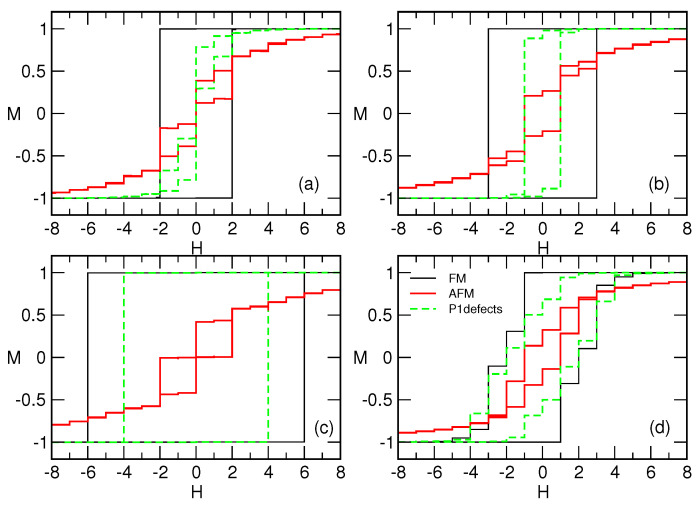
Magnetisation *M* against field *H*, both in reduced units (see text), is shown on different spin networks: grown from mono-dispersive simplexes of the size n= 3 (**a**), 4 (**b**) and 7 (**c**); and for poly-disperse simplexes n∈[2,10] on panel (**d**) for ferromagnetic (FM) or antiferromagnetic (AFM) interactions, and the case of ferromagnetic interactions with antiferromagnetic defects along the topological defect edges. (*p* = 1 for mono-assemblies, and 0.7 for the poly-disperse assembly).

The index i=1,2,⋯,N runs over vertices (nodes) of the network while the index pair (i,j) in the first sum indicates the edges connecting the vertices *i* and *j*. The spin–spin interaction can take a positive or negative sign $J_{ij}/J_{0}=\pm 1$, depending on the pure (ferromagnetic) or defect (antiferromagnetic) edge; $h_{ext}$ is the applied external field. Note that the interaction strength for the exchange-coupled magnetic nanostructures depends on the material and size of nanoparticles and the architecture of the assembly [37]. Theoretical assessment of inter- and intra-particle interactions represents another research direction [36]. In many theoretical studies (see, for example, [39] for Ising modelling of rare-earth tetraborides), the interaction strength is not known, and consequently, the related physical quantities receive numerical values in reduced units. Here, we are primarily interested in describing the impacts of complex network topology and geometric frustration on the spin-reversal dynamics and the hysteresis loop shape. We set $J_{0}=1$ and use the reduced units of the physical quantities. In this type of model, the magnetisation *M* (expressed in Bohr magnetons $\mu_{B}$) is defined as the balance of the number of spins with up and down orientations; normalised by the number of nodes *N*, the dimensionless quantity is limited as $M\equiv \left(N_{↑}-N_{↓}\right)/N\in \left[-1,1\right]$. Meanwhile, the numerical values of the dimensionless field $H\equiv h_{ext}/J_{0}\mu_{B}$ are directly related to the number of nearest neighbours of topologically important nodes; see Figure 3 and the text below.

We apply widely accepted zero-temperature spin dynamics driven by the field (see [46] and references therein). The time *t* measures the number of simulation steps, a step comprising one update of all the spins in the system. The implemented *parallel update* means that the changed state of spin can affect its neighbours only in the next time step. Details of the simulation process are described in the Appendix B; see program flow Algorithm A2. Specifically, starting from a state with all spins down and a large negative field $h_{ext}=-h_{max}$, the field is slowly increased to trigger spin reversals along the ascending branch of the hysteresis. For each particular assembly, the value $h_{max}=k_{max}+\Delta$ correlates with the degree $k_{max}$ (i.e., the number of nearest neighbours) of the leading hub in the network; the field is increased *adiabatically* in small steps Δ until it reaches the other limit $h_{ext}=+h_{max}$. In the present case, due to the fixed strength of interactions, the local field changes in the integer values, which depend on the node’s connectivity. Hence, the spin reversal avalanches are triggered by the external field upon crossing some specific integer values. Following an increase of the external field, the spins are rearranged in an attempt to minimise the energy by alignment along their local fields. The local field $h_{i}^{loc}=-\sum_{j}J_{ij}s_{j}-h_{ext}$ consists of the current value of the external field and the contribution due to interactions with all neighbouring spins. Thus, the flipped spin can cause the changes in local fields in neighbours, causing more flips in the next time step, and so on. The external field is kept fixed until the cascade of spin flips stops, and then it is increased again. Note that the duration of a cascade depends both on the topology and interactions. Consequently, the filed variation in time is highly nonlinear, and can adequately describe the interplay of topology and dynamics, similar to the duration and size of a cascade (avalanche). In contrast to the well-studied random-field ferromagnetic models on different topologies (see [44,46]), we have here the antiferromagnetic interactions along the edges of simplexes, which lead to the frustration effects. Consequently, each spin can not be aligned with its local field to minimise the global energy and alignment of other spins simultaneously. To mimic such situations within the zero-temperature spin dynamics, we allow the spin alignment to occur with a probability *c* smaller than one. A similar approach is used for modelling [47] the domain wall motion during ferroelectric switching [48,49,50].

We find that the magnetisation processes are different, leading to various shapes of the hysteresis loops, depending on the number of antiferromagnetic bonds present and the size of building simplexes, as shown in Figure 3. Notably, for the mono-disperse assemblies (representing the aggregates of cliques of the same size *n*), the rectangular hysteresis loop is found in the ferromagnetic case. At the coercive field $H_{c}=n-1$, related with the vertices of minimum degree, a single system-size avalanche occurs, leading to a complete reversal. In contrast, in the limit with all antiferromagnetic interactions, the loop is slimmer and canted, and never reaches the full-reversal limit. Importantly, the shape of the hysteresis loop crucially depends on the size of the building simplexes. Notably, cf. examples in Figure 3a–c; it splits into a positive and negative loop only when the size of the building simplexes *n* is odd, whereas a central loop with a finite remnant magnetisation occurs when *n* is even. The cases with a finite number of antiferromagnetic defects, i.e., due to the probability p>0 for a defect edge per simplex, the hysteresis loop has some properties of both above cases. In Figure 3a–c, the situation with p=1 is shown for different mono-disperse assemblies. The impact of antiferromagnetic defects again depends on the size of the building simplexes, leading to the reduction of the coercive field to $H_{c}\left(p=1\right)=n-3$. Hence, the severe effects are observed in the case of the assembly of triangles; cf. Figure 3a, where the loops are more similar to the purely antiferromagnetic case. In the case of the assembly of 7-cliques, shown in Figure 3c, the loop is still rectangular, with a properly reduced $H_{c}$ value. The situation with the mixed assembly of simplex sizes n∈[2,10] is shown in Figure 3d. Even though the hysteresis loop in the presence of antiferromagnetic interactions is more narrow compared to the ferromagnetic, in this case, the topological disorder is dominant, resulting in the similar loop shapes.

Another remarkable feature of the hysteresis loop in these structured assemblies is the presence of plateaus at a given level of magnetisation. As also stated in the introduction, a sequence of magnetisation plateaus at fractional values $M/M_{s}$ of the saturated magnetisation $M_{s}$ are experimentally observed in tetraborides and some other disordered antiferromagnetic materials with a strong Ising anisotropy and complex morphology lattices [39,40,41]. Our simulations show that the number and position of these $M/M_{s}$ levels correlate well with the composition of the assemblies regarding the dominant building simplexes. The staircase-like hysteresis loop is present in all types of assemblies with antiferromagnetic interactions. The shape of the loop varies with the size of building simplexes. These findings suggest that the geometrical frustration plays an essential role in these phenomena. Moreover, in the topologically disordered assemblies of mixed cliques n∈[2,10], three prominent plateaus occur due to strictly topological reasons at $M/M_{s}=$ −0.3084, 0.0968 and 0.8518, even in the case of purely ferromagnetic interactions; see the outer loop in Figure 3d. This result implies that a wide variation of the node’s connectivity and the assortative inter-dependence (cf. Figure 2a,b) can lead to a similar phenomenon, even without antiferromagnetic interactions. In this case, we have distinct groups of topologically equivalent spins that respond coherently to the field, resulting in a sequence of steps in the hysteresis loop. This structure thus generalises the rectangular shape with a single step seen in the case of mono-disperse assemblies; cf. Figure 3a–c. Note that these theoretical predictions are based on zero-temperature dynamics and interacting nanoparticle assemblies on a profoundly complex structure. In contrast, many laboratory experiments consider simpler structures or super-paramagnetic assemblies designed for specific applications; see, for example, [51,52,53]. Apart from the finite temperature and a continuous spin symmetry, this topological equivalence can be lifted, leading to a smooth hysteresis loop in the presence of long-range dipolar interactions; the impact of the substrate on which the assembly is realised; and the distributed size of nanoparticles.

**Figure 4:**
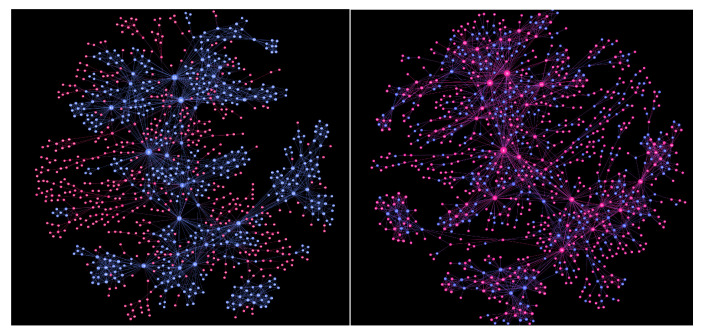
Spin states s=−1 (blue) and s=+1 (red) at the corresponding coercive field $H_{c}=2$ for ferromagnetic (**left**) and $H_{c}=1$ for antiferromagnetic (**right**) interactions on the nanonetwork of polydisperse cliques. The displayed size of vertices is proportional to the number of their nearest neighbours.

Considered on the same structure, the pattern of reversed spins with antiferromagnetic interactions differs significantly from the pattern when the spins have ferromagnetic interactions. For illustration, Figure 4 shows the states of the spins for the corresponding coercive fields in the cases of antiferromagnetic and ferromagnetic interactions, respectively. In the ferromagnetic case when all $S_{j}=-1$, a small positive external field, precisely $h_{ext}=+1$ in the case of mixed assemblies n∈[2,10], is needed to balance the weakest local field, which associates with the least connected nodes. The avalanche then spreads over the nearest neighbour nodes, leading to a jump in the hysteresis loop; cf. Figure 3d. As shown in Figure 4 left, the reversed spins represent connected areas on the graph, in analogy to avalanches in the ferromagnetic RFIM models on regular lattices [46]. In contrast, the reversal in the antiferromagnetic case starts at large negative field values; in this case, a significant positive local field occurs at the nodes with the maximum connectivity and can be balanced by the corresponding values of the negative external field. Thus, the first spin flips occur at the network hubs; they remain surrounded by the spins of the opposite sign, attempting to satisfy all interactions. This situation persists until much larger fields; see Figure 4. According to the network’s assortative nature, spins at next hubs are flipped when the field is further increased. Meanwhile, some spins at small-degree vertices remain unflipped, even at the coercive field, as shown in Figure 4 right. These differences in the reversal patterns are directly manifested in the hysteresis loop shapes discussed above and also affect the course of the magnetisation curves, as discussed in the following.

## 4. The Magnetisation Curves and Multiscale Fluctuations

**Figure 5:**
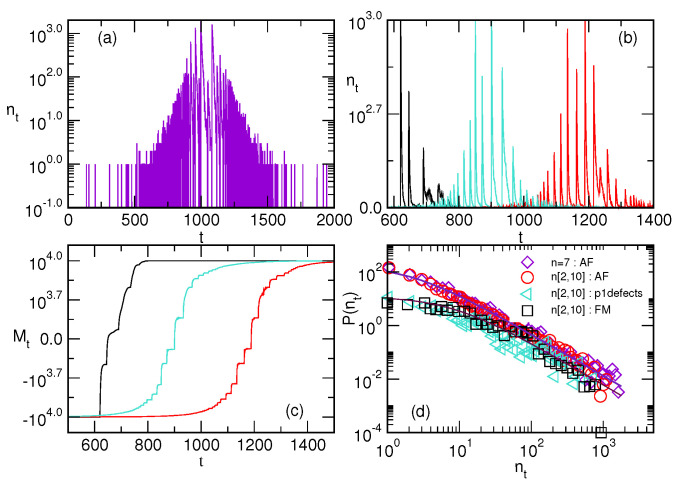
(**a**) The number $n_{t}$ of spin flips per time step *t* in the antiferromagnetic mono-disperse assembly of simplexes with n=7 shows early reversed spins at hubs before the main fluctuations start in the loop centre. For the poly-disperse assembly with n∈[2,10], the signal shapes (**b**), the number of reversed spins $M_{t}=N_{↑}-N_{↓}$ versus time (**c**) and the distribution of the signal $n_{t}$ size (**d**) are shown. Three lines in each panel indicate: ferromagnetic (FM), antiferromagnetic (AFM) and antiferromagnetic defects along defect edges with p=1 (one defect edge per simplex). The distributions in the panel (**d**) are averaged over several realisations; the additional line is for the monodisperse assemblies with n=7 and fully antiferromagnetic interactions.

**Figure 6:**
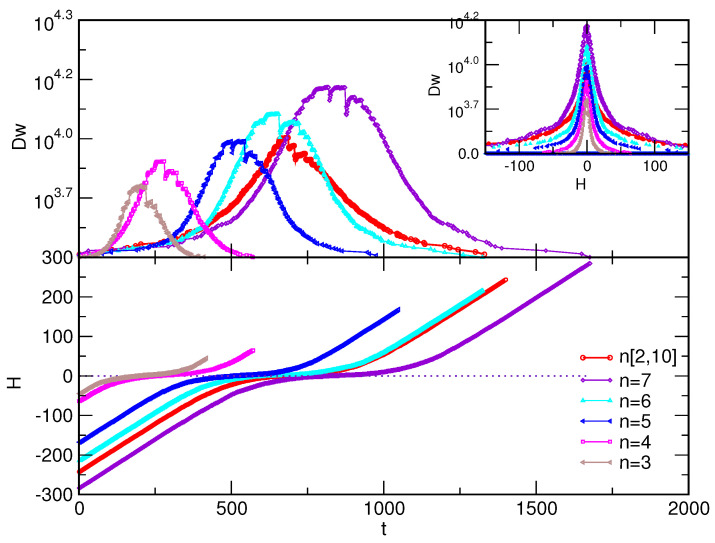
Fluctuations of the domain wall length Dw (in the number of pairs) during the magnetisation process with fully antiferromagnetic interactions against simulation time *t* (top, main panel) and against the external field *H* (inset); the lower panel shows the time variation of the field *H* (in reduced units, see text). The colours/symbols in the legend indicate the different assemblies described in the text.

Figure 5a shows the number of spin flips $n_{t}$ in time for the antiferromagnetic interactions on a monodisperse assembly with the cliques of size n=7. A sequence of single spin flips occurs before the central part of the hysteresis loop is reached, where the corresponding external field raises to near zero. The actual changes of the external field over time for different assemblies are shown in the lower panel of Figure 6.

The main reversal process then occurs in a sequence of events, resulting in the large values of the signal $n_{t}$ around the coercive field, followed by small fluctuations towards the end of the loop. In the ferromagnetic limit, a single large avalanche of spin flips occurs at the coercive field, compatible with the rectangular hysteresis loop in all mono-disperse assemblies, as shown in Figure 3. In contrast, the structured hysteresis loops appear in the case of poly-disperse assemblies, compatible with the fluctuations in a number of spin flips and the corresponding magnetisation curves, as shown in Figure 5b,c. Again, the process is faster and accompanied by a smaller number of avalanches when purely ferromagnetic interactions are considered. The increased number of the antiferromagnetic edges prolongs the process because of the above mentioned different patterns of spin flips and backflips due to frustration. The complete reversal process takes longer in the case of entirely antiferromagnetic interactions, where a small number of spins are active away from the central part of the loop as well. The main part of the process consists of a large number of the active spins, leading to high signal $n_{t}$ of characteristic shape. As the field crosses an integer value in this part of the hysteresis loop, a large number of spins flip, resulting in a high datapoint $n_{t}$ (cf. Figure 5b), followed by a decay where the system stabilises trying to satisfy all the affected bonds. The distribution of the number of active spins per time step, $P(n_{t})$, shows a rather stable power-law tail with the slope close to 1.5, as shown in Figure 5d. The composition of the assemblies contributes to the number of data points with small $n_{t}$ values.

The adiabatic changes of the external field that underlie the magnetisation processes also depend on the structure of the corresponding assembly; the lower panel of Figure 6 shows the field variation with time in the case of fully antiferromagnetic interactions in various assemblies whose structural properties are depicted in Figure 2. The process is accompanied by characteristic fluctuations of the length of the domain wall. Generalising the notion of domain boundary, in the case of network structure, the length of the domain wall is defined as the number of bonds between the spins of the opposite sign. As shown in the top panel and the inset of Figure 6, the domain wall reaches its maximum length when the external field approaches zero. In this region of the field values, the process is prolonged, and significant variations of the domain-wall length occur, suggesting that the frustration effects are at their peak. As Figure 6 shows, these effects are gradually more pronounced with the increased structural complexity of the assemblies, which is enabled by the increased size of the building simplexes.

## 5. Discussion and Conclusions

We have investigated field-induced reversal processes on spin networks composed of geometrically aggregated cliques that enable higher-order connections among spins in the presence of anti-ferromagnetic defects. We have grown networks consisting of the cliques of the same size n=3,4,⋯,7 and poly-disperse assemblies by considering various sizes n∈[2,10] of the building simplexes and a finite probability of a defect edge. In each case, the self-assembly of cliques results in a tree-like pattern of defect edges that carry anti-ferromagnetic coupling between the involved spin pairs. The prominent differences in the structure of various assemblies are characterised by algebraic topology and graph-theoretic measures; additional characterisation for the poly-disperse assemblies with defect edges is provided by topological entropy; cf. Figure 2.

Our results have revealed that the structure of the assembly strongly influences the course of the magnetisation process. Specifically, it depends on the size of building simplexes, the structure of simplicial complexes and the actual pattern of antiferromagnetic defects. The comparative analysis of various mono-disperse assemblies of cliques of the size n=3, 4, ⋯, 7, has enabled us to demonstrate how an antiferromagnetic defect in conjunction with changing higher-order connectivity induces geometric frustration effects. Specifically, for a clique of *n* vertices, enabling an interaction of the *n*-th order, a defect bond causes n−2 frustrated triangles. Thus, the impact of a single antiferromagnetic defect increases with the increased order of simplexes, despite the decreasing relative concentration of defects (p=1 corresponds to 1:n defect bonds). On a larger scale, their influence on the magnetisation process depends on the structure of simplicial complexes in which these frustrated triangles participate.

Considering the shape of the hysteresis loop at the global level, we have shown that severe differences between mono-disperse assemblies occur in the limit of fully anti-ferromagnetic interactions. Not only does the size of the building simplexes play a role, but whether the number of vertices of these building blocks is odd or even does too, as Figure 3 shows. More precise investigations of the pattern of reversed spins suggest that, in the case of anti-ferromagnetic interactions, the reversal starts at the network’s hubs, followed by the next best-connected vertices and so on. In purely ferromagnetic samples, on the other hand, the reversal starts and spreads in a diffusive manner from the least connected vertices. As mentioned in the Introduction, the occurrence of plateaus of fractional magnetisation in anti-ferromagnetic materials on complex lattices is related to the geometric frustration [39,40,41,42]. In our model systems, a sequence of plateaus appears to vary with the size of building simplexes *n* and the actual structure of the anti-ferromagnetic bonds. However, the hysteresis loop with a smaller number of plateaus also appears in the case of poly-disperse assembles with strictly ferromagnetic interactions. These findings suggest that the geometric frustration in these assemblies arises primarily due to structural complexity around the position of the domain-wall.

Our study opens up several questions that require further investigation. These include the analysis of the magnetisation processes when the same building simplexes are self-assembled with a nonzero chemical affinity ν≠0, resulting in different structure of simplicial complexes [3,24], and the multifractal analysis of the magnetisation fluctuations [46,54] that occur in the passages between consecutive plateaus. Further studies are also necessary to investigate the role of higher-order connectivity of these assemblies when the interactions $J_{ij}\in \left[-J,J\right]$ assume random and non-integer values, in analogy to Ising spin-glasses, which are studied on regular lattices [55] and in antiferromagnetic/ferromagnetic bilayers [56]. Another question regards the changes in geometric frustration effects by including the three spin interactions or Heisenberg spins in the Hamiltonian.

In summary, this study reveals the impact that the architecture of simplicial complexes can have on the field-driven spin dynamics. The presence of antiferromagnetic interactions enhances the effects of the geometric frustration in the generalised spin networks with controlled higher-order connectivity. These results can deepen our understanding of the behaviour of complex nanoassemblies, which are currently the focus of materials science and applications.

## Appendix A. Program Flow: Growth of the Graph by Attaching Simplexes with Defects

**Algorithm A1** Program Flow: Growth of the graph by attaching simplexes with defects
1: **INPUT:**$n_{min}$, $n_{max}$, $N_{max}$, *p* 2: initialise graph G as a simplex of size *n* taken from the distribution $p_{n}=An^{-2}$, which is defined in the range $\left[n_{min},n_{max}\right]$ 3: initialise set of defected edges D as an empty set 4: **while** $N<N_{max}$ **do** 5: select new simplex size $n_{new}\in \left[n_{min},n_{max}\right]$ from the distribution $p_{n}$ 6: randomly select the docking site as a simplex σ∈G with folowing constraints: (1) order $q_{\sigma }<n_{new}-1$; (2) σ is not in set of defected edges D; and (3) σ does not contain any edge from D 7: form a new simplex $\sigma_{new}$ by attaching $n_{new}-q_{\sigma }-1$ new nodes to the $q_{\sigma }+1$ nodes of the docking simplex σ 8: **if** random number <*p* **then** 9: **if** σ contains at least one of defected nodes (ends of defected edge) **then** 10: Chose randomly a defected node *a* from σ and randomly *b* as one of $n_{new}-q_{\sigma }-1$ new nodes 11: Add edge (a,b) to D 12: **else** 13: Chose randomly one of newly added edges (a,b) and add to D 14: **end if** 15: **end if** 16: sampling the data of interest; 17: **end while** 18: **END**

## Appendix B. Program Flow: Field-Driven Spin Dynamics on the Graph

**Algorithm A2** Program Flow: Field-driven spin dynamics on the graph
1: **INPUT:** Graph G, store as an array of Edge objects with identity of source and destination vertices, and the edges’s weight wij; the defect edges are marked; the number of all edges is $N_{e}$, the number of all vertices is *N*; input Δ,c≤1; 2: Find $k_{max}$ the maximum number of nearest neighbours among vertices in G; set the external field to $h_{ext}=-k_{max}$; reset time counter; 3: Assign $J_{ij}=-1$ to defect bonds, else $J_{ij}=+1$; 4: Attach two types of spins $s_{i}$ and $m_{i}$ (for parallel update) to each vertex; initialise the spin state as: 5: **for all** vertices i∈G **do** 6: set $s_{i}=-1$ and $m_{i}=-1$; the magnetisation $M=\sum_{i}m_{i}=-N$; 7: **end for** 8: **while** $h_{ext}<+k_{max}$ **do** 9: Field ramping $h_{ext}=h_{ext}+\Delta$ to trigger spin flips; Reset avalanche counters; 10: Flips: reset counter of flipped spins $n_{flip}$; 11: **for all** vertices i∈G **do** 12: compute local field $h_{i}^{loc}$ as the sum $J_{ij}s_{j}$ over all nearest neighbours and add the current $h_{ext}$; 13: check the orientation of the spin $s_{i}$ and the local field and, and with prob. *c*, flip the spin to align it with the local filed; for parallel update, the auxiliary spin $m_{i}$ is flipped; 14: count the number of flipped spins $n_{flip}$; update *M* from the number of flipped $m_{i}$ spins; 15: **end for** 16: update time *t*; sample temporal variables; 17: Swap spins $s_{i}=m_{i}$ at all vertices; 18: **if** $n_{flip}>0$ **then** 19: go to Flips and repeat the process; 20: **else** 21: Determine the avalanche size from the number of flipped spins; 22: **end if** 23: **end while** 24: Sample the data of interest; 25: **END**
